# If There’s Something Strange in Your Neighbourhood, Who You Gonna Call? Perceived Mental Health Service User Suitability for Video Consultations

**DOI:** 10.3390/healthcare9050517

**Published:** 2021-04-29

**Authors:** Jon Painter, James Turner, Paula Procter

**Affiliations:** Department of Nursing and Midwifery, Faculty of Health and Wellbeing, Sheffield Hallam University, Sheffield S1 1WB, UK; James.turner@shu.ac.uk (J.T.); p.procter@shu.ac.uk (P.P.)

**Keywords:** telehealth, telepsychiatry, video consultation, Skype, mental healthcare, nursing

## Abstract

COVID-19 has placed additional challenges on mental health services. Video consultations (VCs) have provided a short-term solution to lockdown restrictions but could also increase long-term capacity to meet the anticipated rising demand. A total of 7752 VCs were conducted over six weeks. Thematic analysis of 474 online survey responses identified twenty patient attributes that influenced staffs’ decisions to offer VCs. Their opinions were diverse, at times contradictory, and not always evidence based. There was reasonable consensus (and published evidence to support) of the probable suitability of VC for patients who: are IT savvy and suitably equipped; are teenagers; live in remote/rural locations; have caring responsibilities; have anxiety disorders or express a preference. No consensus was reached regarding eight attributes and there was a corresponding paucity of evidence, indicating the need for further research. Conversely, old age; paranoia, sensory impairment/communication difficulties; high risk and trauma/PTSD (posttraumatic stress disorder) were generally seen as contraindicated by staff, despite published evidence of success elsewhere. It may be possible to overcome staff’s reticence to offer these groups VCs. As staff are effectively the gatekeepers to VC interventions, it is important to understand and support them to overcome reservations that are contrary to the empirical evidence base. This will ensure that their initial anxieties do not become unnecessary barriers to services for those most in need. As with all mental healthcare, such decisions should be made collaboratively, and on an individual basis.

## 1. Introduction

The phrase “strange and unprecedented” seems to have become ubiquitous in most discussions about healthcare during the COVID-19 global pandemic. Mental healthcare in the United Kingdom has been under considerable strain for over a decade [1]. Recently, this pressure has increased further due to the challenges of continuing to provide care/treatment during COVID-19 lockdown restrictions [2]. In the future, as the psychological impact of COVID-19 (and long COVID-19) plays out, it is anticipated that the demand for these services will dramatically rise again [3,4,5,6].

In response to the long-standing cost pressures, some mental health providers have already turned to video consultations in an attempt to gain efficiencies and ‘do more with less’ [7,8]. Since COVID-19, many more providers have moved some or all of their services online [9,10,11]. However, unlike many other fields of healthcare, mental healthcare is as much an art as a science [12], with an effective therapeutic alliance an essential pre-requisite [13,14]. Although video calls in everyday life have become increasingly routine, they remain, for many patients, relatively novel and, for some, a thoroughly disconcerting experience [15,16] that could theoretically threaten the development and maintenance of therapeutic relationships with healthcare professionals.

Rhetoric would suggest that certain groups (such as older people) struggle to adapt to technology in general but, in mental healthcare, there are numerous examples of effective online treatment programmes for older adults [17,18]. This highlights a dissonance between preconceptions and the reality of who may or may not be suitable to receive mental healthcare via synchronous video calls. As staff are effectively the gatekeepers to this form of treatment, their preconceptions are important as they could pose barriers to mental healthcare for certain vulnerable groups of patients at a time when they most need services [1,19], thus creating damaging self-fulfilling prophecies. In light of these issues, this paper reports the relevant findings from one large mental health and disability NHS trust’s video consultation (VC) project that started before, and extended into, the current COVID-19 pandemic. The project’s overarching aim was to increase efficiency by reducing the amount of time staff and service users spent travelling to appointments whilst maintaining their levels of satisfaction.

## 2. Materials and Methods

The overall project combined routinely collected data from several trust databases with staff and service user feedback. These data were gathered between 1 June and 15 July 2020 as part of its informatics team’s ongoing monitoring of their VC software’s implementation. To maintain confidentiality and GDPR compliance throughout the project, all data were collated and pseudonymised by the trust’s informatics department before being encrypted and securely transferred to the university for storage and analysis on its dedicated, password-protected, research servers. Consequently, this study was defined (and registered) as a naturalistic retrospective, service evaluation by the trust’s research department (SER-19-019—NTW) and ethically approved by the university (ID: ER15924620).

With reference to the literature [20], and the project brief, a bespoke set of questions was developed as an online staff survey (Table S1, supplementary material), two of which are the subject of this paper:

1. “Are there any types of service user who would be particularly suited to online video consultations and why?”
2. “Are there any types of service user who you would not offer online video consultations and why?”

The survey was circulated by email twice (mid-June and mid-July 2020) to all staff registered to use the video-consultation software at each point in time. Completion was voluntary and, to maximise the chances of candour, staff could also choose to respond anonymously.

Once responses to the two questions were received, separate thematic analyses were conducted using Braun and Clarke’s [21] six-phase inductive approach. This includes (1) familiarisation with data, (2) coding of the data such that the entire dataset is reviewed and each piece of relevant text (data) is tagged with a code, (3) consideration of themes, (4) revision of themes, (5) analysis of individual themes and (6) write up.

A theme was noted when codes related to the research question arose that had a recurring pattern in the data. During stage five of the analyses [21], the two individually generated sets of themes (i.e., patient types/attributes) were reviewed and it became clear that there was considerable similarity. Therefore, in the interests of parsimony, themes from each question were adjusted and aligned to form a single set of themes/attributes.

Finally, results were tabulated, with the staff’s arguments for and against each patient type/attribute summarised and augmented by respondents’ illustrative verbatim quotes. This was intended to highlight the considerable variation and, at times, contradictory views that were expressed.

## 3. Results

Over the six-week data collection period, 7752 VCs were conducted by staff from 137 clinical teams/services that, collectively, covered all age groups and ‘types’ of service user served by the trust. In June 2020, 468 staff made video calls. Survey responses were received from 264 of these staff (response rate = 57%), of which 32 were anonymous. In July 2020, 558 staff made calls and a further 216 responses were obtained (response rate = 48%), of which 31 were anonymous.

In total, there were 474 responses to the question:

- “Are there any types of service user who would be particularly suited to online video consultations and why?”

and 477 responses to the question:

- “Are there any types of service user who you would not offer online video consultations and why?”

The themed responses (i.e., patient groups/attributes) have been synthesised, ranked, tabulated and emboldened in Table 1 to highlight the number and nature of the many different, and often contradictory, opinions that were provided. Bold text indicates that there was a consensus of at least 70% of the responses for that theme/patient group.

N.B. Patient attributes with less than 20 responses in total were deemed insufficient to draw anything but tentative conclusions and hence were left unshaded.

## 4. Discussion

The tabulated results above are not intended to form any sort of crude suitability checklist. Instead, they illustrate the (often differing) opinions that staff held regarding the suitability of different patient groups/attributes. In this regard, they highlight the multi-facetted clinical judgement needed to determine an individual patient’s suitability for VCs. However, it is important to consider the validity of these perceptions as they may or may not be evidence based. To facilitate this, a brief, desktop search of the literature was undertaken (see also Figure S1, supplementary material).

### 4.1. Perceived Suitability

The staff’s consensus for seven of the twenty emergent themes (i.e., 1, 2, 3, 4, 7, 11, and 12) was that these types of patients were likely to be well suited to VCs. As in Feijt et al.’s study of staff’s perceptions [22], IT literacy and access to appropriate equipment were deemed essential. In 2016, Leigh [23] estimated that 71% of Britons owned a smartphone, and elsewhere, 66–75% of people with serious mental illnesses were thought to be IT literate [24]. As these figures are only set to rise, the proportion of suitably equipped and able psychiatric patients will helpfully increase. An expressed preference was arguably seen as the most significant factor and, although there may be times when patients make treatment choices that are not in their best interests, this is certainly in keeping with the notion of patient-centred care and “No decisions about me without me” [25].

Unsurprisingly, the convenience of VCs for people residing in remote/rural locations was recognised by staff and is well established in the literature [5,13,26,27,28]. However, (although arguably less relevant to the UK) there are cautions/caveats regarding online consultations that cross state/legislative boundaries [22]. Similarly, where larger geographical catchment areas are created, online services must remain culturally sensitive [29]. The convenience of this treatment modality was also recognised for patients with caring responsibilities, something that again repeatedly occurs in the literature [30,31,32], albeit with Payne et al.’s warning to ensure confidentiality is maintained during calls to busy/chaotic households [13].

Whilst staff generally saw the next three patient groups as suitable, unlike those discussed to this point, some counter-arguments were also made (a pattern replicated in the published literature). Staff generally felt that younger people were well suited to VCs [27,29], but that there were some exceptions (e.g., the very young and those with concentration difficulties). Their reservations chime with Payne et al., who found insufficient published evidence for VCs with young people experiencing Attention Deficit Hyperactivity Diaorder (ADHD) and Autistic Spectrum Disorder (ASD) [13]. Similarly, although video calls to those experiencing anxiety, avoidance and/or dissociation were generally seen as helpful, some staff had reservations about their merits. In studies elsewhere, Chen et al. supported their use in treating anxiety disorders including agoraphobia and Obsessive Compulsive Disorders [30]. However, conversely, Valentine et al. detected lower treatment retention rates in US war veterans with anxiety [33]. Interian et al.’s findings support both notions, i.e., that, although they can be useful when starting to build a therapeutic relationship, they can become counter-productive if leaving the house is an identified treatment goal [34].

### 4.2. Uncertain/Disputed Suitability

For eight of the emergent patient groups/attributes (i.e., 5, 6, 9, 10, and 16), there was no clear staff consensus or too few views expressed to reasonably draw firm conclusions (i.e.,18, 19, and 20). Some of these themes (e.g., “unsure”) are not easily compared to the literature. Some (e.g., “physical health needs”) are too broad to yield a single answer; and for others, there is, as yet a paucity of evidence. However, for three, there is at least some evidence worthy of comparison to the staffs’ opinions.

Views on the suitability of VCs for people with learning disabilities, ASD and/or ADHD did not quite reach the 70% threshold set to constitute a consensus but, 69% of staff reportedly rejected them in favour of in-person contacts. In contrast, acknowledging that the evidence base is inconclusive, Hollis et al. found some empirical evidence of successful delivery of online interventions to young people with learning disabilities [35]. Similarly, Shore et al. also provide some compelling evidence via a case study of a child with Asperger’s Syndrome who was able to engage more effectively when physically distanced from his therapist, suggesting that VCs should not be dismissed out of hand for people accessing a range of learning disability and Autism services [36].

Lin et al. found that health centres with large homeless populations were less likely to employ online technologies [28], perhaps due to the associated issues of poverty [37]. More positively, Payne et al. found that men (traditionally reluctant help-seekers) often seemed more candid online [13] and Lauckner and Whitten found that veterans (another reticent patient-group) engaged well with VCs [27]. Only eight staff comments were grouped under the theme of “hard-to-reach patients” but all were positive about the potential for online working with this group, suggesting definite growth potential.

Finally, although small in number, there were suggestions that the delivery of formal talking therapies via video calls breached some professional bodies’ expectations/regulations. Given the number of published studies of online therapies, this is an unexpected finding. However, for online therapy to flourish, staff need professional and managerial reassurance, appropriate regulation and clear policies to feel adequately secure [5,38,39].

### 4.3. Perceived Unsuitability

Finally, VCs were seen by staff as largely contraindicated for five of the emergent categories of patient (i.e., 8, 13, 14, 15, and 17). Perhaps the most obvious of these is patients experiencing paranoia, or who are particularly suspicious of technology/authority [30]. Clearly, any treatment modality that exacerbates an individual’s problems should generally be avoided. However, like Shore et al. [7], some staff felt that there might be a subset of these patients for whom the graded use of VCs could, over time, help challenge these distressing thoughts and emotions.

Another group that staff were reluctant to engage with online were patients labelled as high risk, a phenomenon also highlighted by Pierce et al. [32]. As with physical health problems (discussed earlier), this theme is relatively broad. Some risks, e.g., suicidality, may be mitigated with robust protocols for staff working by video [5,29] but others, e.g., aggression to staff [40] or COVID-19 transmission (as suggested by staff), may actually be reduced by its use. This suggests that the nature as well as the level of risk should be considered on a case-by-case basis. However, it also raises the possibility that risk is actually a proxy for some other (underlying) factor. A common concern expressed by staff is the limitations that VCs place on their ability to distinguish and interpret non-verbal and paraverbal cues [7,22]. It may be that, together with a perceived lack of managerial support [38], staff feel professionally vulnerable working in this new (and arguably limiting) assessment medium. To support this notion, sensory impairment and communication issues emerged as a theme in this staff survey, as well as the literature [30]. However, it is again important not to immediately dismiss all such patients as simple solutions such as headphones and the careful setting up of equipment can help ameliorate many difficulties [29].

In a similar vein, 98% of the responses relating to older adults were doubtful of the merits of VCs. However, the theme also included the closely related issue of cognitive decline/impairment. It is therefore, again, possible that age was a proxy for a range of other, age-associated, complexities that require a more nuanced assessment. This stance is supported by literature, some of which suggests that older people are unsuited to this technology [26], that there is a clear digital divide [30], and consequently that it is used less by older people’s services [28]. However, conversely, there are other studies reporting that age has little or no bearing [33] and offering examples of successful video consultation services with older adults [7,17,18]. Either way, age is likely to become an increasingly moot point as the proportion of tech-savvy older people inexorably increases [41].

The last patient group to thematically emerge from the survey responses related to those who had been traumatised and/or were experiencing PTSD. Here, 80% of responses indicated that staff thought VCs were inappropriate. However, there is a reasonable amount of literature to challenge this [27,29,33] as well as Kruse et al. [26], who reported that military veterans with PTSD found the technology easy to use, contradicting not only staff’s perceptions regarding trauma/PTSD but potentially age as well.

## 5. Conclusions

Against the backdrop of COVID-19, technologies such as VCs have reduced many of the barriers for people requiring access to mental health services [26]. However, uptake remains limited [7]. Mental health service staff are effectively the gatekeepers to support/treatment as they largely dictate who is offered an online alternative to an in-person intervention [27]. Many are apprehensive and reluctant to adopt this new treatment modality [5] but the precise reasons are hard to pin down [22] and under-researched [38] and when they are, studies are often limited to listing potential factors with no quantification/ranking [22].

A key step in increasing the uptake/use of VCs is to address the myths, preconceptions, and misconceptions that staff hold by referring to the evidence [40]. Therefore, this study sought to both theme and quantify staff’s perceptions of suitability before considering these results in light of the literature. Twenty patient types/attributes were identified from 951 responses to an online staff survey. A consensus emerged for twelve but only six conclusions were unanimous with differing levels of counter-arguments raised for the other six. A similarly disparate pattern was apparent from even a cursory search of the literature, meaning that most staff perceptions could be supported by at least some empirical evidence.

Here it is important to acknowledge the limitations of this study, i.e., that the literature search/review was far from systematic (as defined by Grant and Booth [42]); that the staff were a convenience sample from whom no socio-demographic data were gathered; that the overall response rate was 52%; and that, at the time of responding, staff had differing levels of experience with VCs. In addition, the method of quantifying their perceptions was rudimentary. That said, the number of responses was reasonable and spanned a wide variety of clinical teams/services, giving this naturalistic study good ecological validity.

Overall, it seems reasonable to conclude that, whilst many of the staff’s instincts are justifiable, at least some might reasonably be challenged by referencing the growing number of empirical studies. This should, however, be done in a supportive manner [32] so that staff are left feeling reassured, rather than unduly pressurised as this would be counter-productive and potentially lead to the transference of their anxieties to their patients [7].

COVID-19 has, in many ways, acted as a catalyst for a change that was both inevitable and already underway. However, from this study, it seems that, if there’s something strange in the neighbourhood, it is important to support staff in deciding who they are gonna call [43].

## Figures and Tables

**Table 1:** Patient types/attributes perceived by staff as suitable/unsuitable for video consultations.

Theme No.	Patient Type/Attribute	Number of Comments and Any Rationale re. Suitability for Video Consultations	Number of Comments and Any Rationale re. Unsuitability for Video Consultations
1	IT literate and with suitable equipment (*n* = 136)	**100% (*n* = 66). Tech-savvy patients with access to suitable equipment, stable Wi-Fi, and sufficient data. “*Patients with a computer that cannot attend the clinic in person for any reason*”. (R26) N.B. Younger people were perceived to meet these criteria most frequently. “*Teen age group, very used to interacting in this way*”. (R43)**	0% (*n* = 0). N.B. Some responses suggested that older people, in particular, would struggle in this regard. “*Older people who may not be adept at using technology or devices*”. (R153)
2	Anxious/avoidant/dissociative (*n* = 106)	**85% (*n* = 90). 18 staff advocated video calls for the initial assessment/brief interventions for patients with anxiety, PTSD (posttraumatic stress disorder) or GAD. Agoraphobia was singled out on several occasions, e.g., “*Initially I think for therapy…I would want to progress to face-to-face, but this could be an option for starting*”. (R415) They “*would want them to get out eventually*”(R236) *… so as not to* “*collude with avoidance of feared situations*”. (R327) Other comments included “*Socially anxious clients, those with OCD who are concerned about contamination—coming out of their home. Those with health anxiety and those shielding during lockdown*”. (R320) “*People with mild and less complex presentations of depression and anxiety people who are not yet comfortable coming to face to face sessions*”. (R112)**	15% (*n* = 16). 16 argued that those experiencing anxiety would find it hard to engage, as would those who were dissociative as it “makes them hard for them to feel present”. (R223)
3	Expressed preference (*n* = 101)	**100% (*n* = 101). Rather than any particular type of patient, any patient preferring face-to-face intervention should be accommodated on a case-by-case basis. “*Any—I don’t think we should make assumptions; this should patent/service user and carer lead*”. (R362)**	0% (*n* = 0). No counter-arguments were expressed.
4	Teenagers/younger adults (*n* = 89)	**74% (*n* = 66). Older teenagers, students, and young adults “*are from that generation of people who have been brought up with technology and therefore this is easily accessible for them*”. (R289)**	26% (*n* = 23). Young children (especially if hyperactive) “find it hard to focus” and “find it hard to sustain interest remotely”. (R134)
5	All/none (*n* = 89)	66% (*n* = 59) of staff suggested all types of patients may be suited and they should be “*an option for all*” (see also expressed preference comments). (R138)	26% (*n* = 20) of staff felt that no patients (or very few) would be suitable for video consultations. “*I can’t think of any*” (R38)
6	Unsure (*n* = 79)	65% (*n* = 51) of responses regarding suitable patients were blank, marked as N/A or unsure. “*Can’t think of any specifically*” (R57)	35% (*n* = 28) of responses regarding unsuitable patients were blank, marked as N/A or unsure.
7	Living in isolated, rural settings and/or far from staff bases (*n* = 72)	**100% (*n* = 72). “*Rural patients without good access to transport. Often these patients are disadvantaged and have to spend long periods of time on public transport for a relatively short appointment; if a proportion of their appointments could be done remotely this would save them time and also perhaps childcare issues*”. (R37) The issue of the time/financial burden of travel was particularly pertinent for services with a national remit i.e., “*those outside regional ‘catchment’*”. (R137)**	0% (*n* = 0). No counter-arguments were expressed.
8	High risk to self/others (*n* = 67)	10% (*n* = 7). Patients with a history of violence were mentioned most often here, e.g., “*The ones who are shielding*”; “*similarly patients who have risk of violence can be assessed via online consultation*”. (R342) “*Less need for two person assessments where there are potential risk issues to staff*” (R12) An innovative suggestion was community in-reach to wards when “*clients are in seclusion with safety issues*”. (R129)	**90% (*n* = 60). Many staff prefer face-to-face contact “*if they* (patients) *are of high risk of self-harm behaviours*” (R457), “*harm to others*” (R226), in “*crisis*” (R52), are “*impulsive*” (R203), “*experiencing extreme distress and aggression*” (R454) or are “*acutely unwell*” (R227). Vulnerable patients were also cited, i.e., the homeless, victims of abuse and those subject to safeguarding as confidentiality could not be guaranteed “*because* [it is] *harder to assess and manage risk when not in a room with them*” (R159).**
9	Learning disability/Autistic Spectrum Disorder (ASD)/Attention Deficit Hyperactivity Disorder (ADHD) (*n* = 65)	31% *(n* = 20) of staff expected (or had experienced benefits) for patients with learning disabilities in general, and ASD specifically, e.g., “*many service users (suspected autism) prefer to utilise* (video) *for assessment over leaving home to attend a face-to-face appointment in clinical workplace*” (R3) and “Autistic people all enjoyed maintaining contact especially to reduce anxieties when first admitted to the unit”. (R234)	69% (*n* = 45) of responses included 20 about patients with learning disabilities struggling, especially without support to use the software. Others suggested “*there seems to be a pattern emerging in our young people with a neurodevelopmental profile (e.g., ASD, ADHD) that have requested not to have online video consultations and are preferring to wait until face-to-face is permitted*” (R289). Patients with ASD “*find it extremely difficult to function well on online consultations*” (R250). “*Children with ASD are tricky to capture on screen if they do not want to engage it is more forced online*”. (R382*)* One simply said, “*not in LD services*”. (R421)
10	Physical health needs (*n* = 61)	61% (*n* = 37) of comments related to long-term physical health issues, frailty, mobility problems and shielding from COVID-19. Post-pandemic, patients who “*otherwise would miss out on therapy … and assessments*” (R469) were identified as being particularly suitable.	39% (*n* = 24) of staff identified several physical interventions that were not viable by video, i.e., physical examinations/vital signs monitoring, dysphagia assessment, depot injections, and urine testing. Additionally, higher-level MSE examination or cognitive assessment, “*where language intonation, breathing rate, levels of stress etc. are key to understanding presentation*” (R61) were deemed problematic.
11	Family/caring responsibilities (*n* = 45)	**100% (*n* = 45). In addition to patients requiring formal family work, “*I predominantly work with client’s who have care responsibilities, this medium helps manage their care obligations and still attend therapy which was not always the case with face-to-face appointments.*” (R520) This was especially true for (shift) working parents who may “*wish to have treatment within a small time window e.g., their lunch hour*”. (R397) It was also suggested that “*perinatal ladies would benefit*” (R65) in particular.**	0% (*n* = 0). No counter-arguments were expressed. However, caveats noted elsewhere (e.g., noisy homes) should be borne in mind.
12	Settled and/or well-known patients (*n* = 43)	**74% (*n* = 32). “*Settled patients, who have an established rapport with staff that know them well and have already been seen face-to-face were identified as suitable for video consultations*”. Examples of mid-therapy interventions viable by video with low-risk, low-complexity patients included general monitoring, medication reviews, exposure therapy, coping strategy enhancement. “*It’s probably better to use for established relationships and for more practical therapy tasks*”. (R48)**	26% (*n* = 11) of respondents believed that engaging and assessing new patients should be face to face, e.g., “*might be better for people who I know rather than new people*”. (R50) Also, there was concern that the “*Association of Family Therapy does not recommend meeting with new families via video calls*”. (R306)
13	Cognitive deficits/older people (*n* = 43)	2% (*n* = 1). Respondent was “*very pleasantly surprised how many older folks are also finding it helpful*”. (R61)	**98% (*n* = 42) of comments were related to either cognitive deficits (*n* = 24) or older people (*n* = 18). Although captured as separate nodes, the degree of overlap warrants their amalgamation here. Areas of concern were unfamiliarity/complexity of technology for older people, especially those lacking capacity, and diagnosed with dementia or other neurological deficits. “*Elderly patients without support if there are concerns about capacity, consent or confidentiality.*” (R193)**
14	Psychosis/paranoia (*n* = 33)	24% (*n* = 8). “*Paranoid patients may be too fearful to attend hospital premises*” (R385) “*Most are, in the psychosis service, however some have preferred to not have the video on to avoid eye contact. In real life they would otherwise not attend or become hostile*”. (R90)	**76% (*n* = 25). Actively psychotic or paranoid patients (especially with worrying beliefs about technology) would have concerns about privacy and experience trust issues. “*Paranoid patients can worry about sessions being recorded*” (R22)**
15	Communication difficulties/sensory impairments (*n* = 31)	0% (*n* = 0). Although offering definite advantages over phone calls, there were no comments advocating video consultations for patients with communication difficulties.	**100% (*n* = 31). There were 15 comments about patients with specific sensory “*hearing/sight impairments*” (R200) who potentially require interpreters. A further 16 comments related to communication problems more broadly, e.g., noisy homes, distracted parents, shyness, where “*English is not their first language*” (R130) and “*those who are dysphoric regarding their image and/or voice where video consultations can result in them seeing and hearing themselves which may not be helpful for them in engaging with therapy*”. (R135)**
16	Complex dynamics (*n* = 22)	41% (*n* = 9). Video was potentially helpful where multiple professionals, paid carers and/or parents were required to collaborate on a particular patient’s care/treatment. “*Really helpful for working with families as telephone consultations are limited in usefulness*”. (R199) “*Very good for case reviews as can have more than 1 professional attending*” (R201)	59% (*n* = 13) gave examples of circumstances/traits that could be complicated by the use of video included institutionalised patients, over-dependence, attachment issues, passivity, avoidant, excessively anxious, self-consciousness and “*patients who try to hide their symptoms.*” (R437) There was also one suggestion that the dynamics of “*family therapy is difficult to complete due to ethical and safeguarding concerns*”. (R306)
17	Trauma/Post Traumatic Stress Disorder (PTSD) (*n* = 20)	20% (*n* = 4). “*Trauma clients who are reluctant to go out*” (R322) could benefit from video consultations.	**80% (*n* = 16). Video consultations may be too intense for some trauma clients. Call drop out mid-disclosure could also be damaging. “*I would consider most trauma focused therapy risky or unhelpful via online consultation as it is helpful to be in the same room to both pick up subtle difficulties/symptoms someone may be showing (that would be difficult to pick up online) but also support clients if they become significantly distressed/dissociate/etc.*” (R195)**
18	Specific therapies Cognitive Behaviour Therapy/Eye movement Desensitisation Therapy/Dialectic Behavioural Therapy(CBT/EMDR/DBT) (*n* = 12)	92% (*n* = 11). There were examples of formal therapy sessions being successfully delivered via video CBT (*n* = 6), DBT (*n* = 3), and EMDR (*n* = 2), e.g., benefiting from the additional structure this provided or “*if they needed a quick DBT recap for relapse prevention*” (R219)	8% (*n* = 1) of staff commented that CBT by video was problematic as sessions could not be recorded which was “*not in line with BABCP accreditation processes*”. (R392)
19	Hard to reach (*n* = 8)	100% (*n* = 8) of staff cited cases where hard-to-reach patients (e.g., poor attendees, school refusers, homeless, sofa-surfers, and “*hard to engage adolescents*”) (R35) had engaged more reliably via video than face to face.	0% (*n* = 0). No counter-arguments were expressed.
20	Eating disorders (*n* = 3)	0% (*n* = 0). No counter-arguments were expressed.	100% (*n* = 3) of responses noted that video may not be suitable for patients with eating disorders who “*dislike seeing themselves*” or where “*weighing in session is an important part of most treatment*”. (R399) (See also physical health needs above.)

Bold = consensus view of the staff (consensus of at least 70%); ASD = Autistic Spectrum Disorder; ADHD = Attention Deficit Hyperactivity Disorder; PTSD = Post Traumatic Stress Disorder; CBT = Cognitive Behaviour Therapy; EMDT = Eye movement Desensitisation Therapy; DBT = Dialectic Behavioural Therapy.

## Data Availability

On request from the corresponding author.

## Supplementary Materials

The supplementary materials are available online at https://www.mdpi.com/article/10.3390/healthcare9050517/s1.
