# Supplementary material for: If There’s Something Strange in Your Neighbourhood, Who You Gonna Call? Perceived Mental Health Service User Suitability for Video Consultations
Source: Healthcare (Basel). 2021 Apr 29;9(5):517. doi: 10.3390/healthcare9050517 (PMC8146145; doi:10.3390/healthcare9050517)
Supplement: Supplementary file 1 [file healthcare-09-00517-s001.zip › healthcare-1157922-supplementary.pdf]

healthcare

MDPI

Table 1. Copy of online staff survey questions.

Question	Response options
1) Please enter your name (or leave blank if you prefer to complete the survey anonymously)	Free text
2) Please select the date of completion of this survey using the calendar icon	Calendar icon
3) Please enter the locality and service you work in.	Drop down option list
4) Thinking about using online video consultations in the future, how likely are you to use online video consultations as part of service users treatment if it were available?	Extremely likely/ likely/ neither likely nor unlikely / unlikely / very unlikely
5) How likely are you to recommend using online video consultations as part of a service user's treatment to other members of your team?	Extremely likely/ likely/ neither likely nor unlikely / unlikely / very unlikely
6) Please use the box below to detail any benefits you gained from using online video consultation?	Free text
7) What were the least helpful aspects of using online video consultations?	Free text
8) Are there any types of service user who would be particularly suited to online video consultations and why?	*Free text*
9) Are there any types of service user who you would not offer online video consultations and why?	*Free text*
10) Please rate your overall experience of using online video consultations?	Poor / fair / moderate / good / excellent
11) Using an online video consultation allowed me to interact with service users the same way as you would have in a face to face consultation (in person)?	Strongly agree / agree / disagree / strongly disagree
12) I feel comfortable communicating with service users while using an online video consultation?	Strongly agree / agree / disagree / strongly disagree
13) If a service user became anxious or distressed during the online video consultation, using online video consultation hampered my ability to manage the situation?	Strongly agree / agree / disagree / strongly disagree
14) I was able to detect changes in a service user's voice, mannerisms or facial expressions during an online consultation?	Strongly agree / agree / disagree / strongly disagree
15) How would you rate the audio quality of the software?	Poor / fair/ moderate / very good / excellent
16) How would you rate the video quality of the software?	Poor / fair/ moderate / very good / excellent
17) How would you rate the "ease of use" of the software?	Poor / fair/ moderate / very good / excellent
18) If you encountered any technical issues while using the software, please use the text box to give details of the issue and indicate if this was reported and resolved by the Informatics Project …	Free text
19) When Covid 19 restrictions end, what proportion of your service user contacts do you think will still be online? Please give an estimated percentage in the box	Free text

N.B. Question 8 and Question 9 are the focus of this paper.

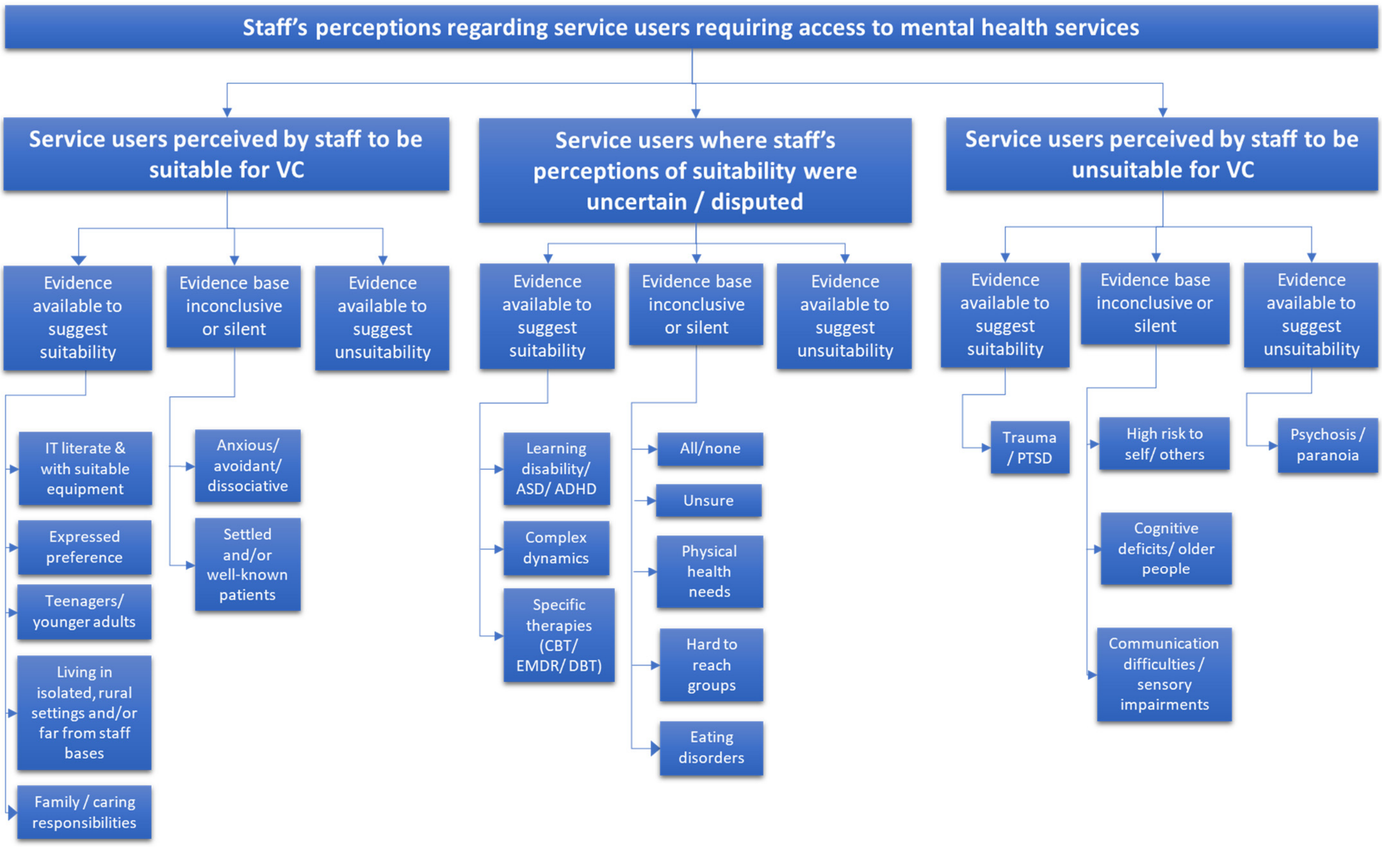

Figure S1. Coding tree.
